# Towards health equity for people experiencing chronic pain and social marginalization

**DOI:** 10.1186/s12939-021-01394-6

**Published:** 2021-02-02

**Authors:** Bruce Wallace, Colleen Varcoe, Cindy Holmes, Mehmoona Moosa-Mitha, Gregg Moor, Maria Hudspith, Kenneth D. Craig

**Affiliations:** 1 School of Social Work, University of Victoria, PO Box 1700, STN CSC, BC Victoria, Canada; 2 Canadian Institute for Substance Use Research (CISUR), P.O. Box 1700, STN CSC, BC V8W 2Y2 Victoria, Canada; 3 School of Nursing, University of British Columbia, T153-2211 Wesbrook Mall, BC V6T 2B5 Vancouver, Canada; 4 Pain BC, 1508 W Broadway, V6J 1W8 BC Vancouver, Canada; 5 Department of Psychology, University of British Columbia, 2136 West Mall, BC V6T 1Z4 Vancouver, Canada

**Keywords:** Chronic pain, Health equity, Social marginalization, Structural violence, Community‐based participatory research (CBPR), Indigenous, LGBTQ2S, Refugee

## Abstract

**Objective:**

For people who experience social inequities and structural violence, pain and related care are inexorably linked to experiences of injustice and stigma. The purpose of this study was to examine in greater depth the experiences of pain and discrimination and stigma across diverse marginalized communities in order to recommend equity-oriented healthcare approaches.

**Methods:**

This community-based qualitative study reports on four focus groups that included 36 people living with pain. All participants identified with one of three groups known to experience high levels of inequities and structural violence including an Indigenous group, a LGBTQ2S group, and two newcomer and refugee groups.

**Results:**

Pain was entangled with and shaped by: social locations and identities, experiences of violence, trauma and related mental health issues, experiences of discrimination, stigma and dismissal, experiences of inadequate and ineffective health care, and the impacts of these intersecting experiences.

**Conclusions:**

Equity-oriented responses to chronic pain would recognize pain not only as a biomedical issue but as a social justice issue. The EQUIP Framework is an approach to integrating trauma- and violence-informed care; culturally-safe care; and harm reduction in health care that may hold promise for being tailored to people experiencing pain and social marginalization.

## Introduction

Chronic pain is a major public health challenge, as reflected in widespread prevalence, seriously debilitating impact, and resistance to existing treatments [1, 2]. The biopsychosocial conceptual approach is widely endorsed in medicine and provides a guiding framework for understanding pain [3, 4], but there are disproportionate emphases in application of components of the model, with everyday treatment firmly focused on biomedical care, despite substantial evidence for the efficacy of psychosocial interventions [5]. Social parameters governing access and delivery of care receive relatively little attention and remain poorly understood.

The importance of the social context for understanding and treatment of chronic pain is revealed in recent findings indicating that persistent pain is often poorly recognized [6–8], under-estimated [9–11], and inadequately managed [12, 13] in the general population. This is accentuated by substantial disparities in care across population groups – particularly in marginalized populations who are more likely to be compelled to live with pain and pain that is of greater severity [1, 14]. This contrasts unfavorably with declarations that pain management is a fundamental human right even tough inequities and injustices are identified as the greatest challenges to responding to pain globally [15]. Thus, there is value in theoretical models of pain that attend to social determinants of pain [16–19].

Central to understanding the limited care options available to people experiencing chronic pain is evidence that chronic pain is associated with social stigmas and discrimination [19]. This intersects with multiple stigmas related to race and ethnicity, class, gender, sexual orientation, mental health, and substance use, among other forms [16–19]. Thus, chronic pain becomes more than a health issue—it should be viewed as an issue of equity and justice associated with social contexts of discrimination and structural violence [20], reflective of the way in which societies are organized to create harm and maintain racism, poverty, and other disadvantages [21]. While physical trauma in the form of injury and surgical interventions represents the major cause of pain, social trauma resulting from experiences and impacts of racism and colonization [22, 23], the stigma and lack of responses for mental health issues [24], misogyny and intimate partner violence [25], the current drug overdose public health crisis [26] [27], and the ongoing crises of homelessness and poverty [28], among other challenges, contribute substantially to experiences of living with pain. Therefore, paying attention to the social determinants of pain is crucial to realizing a more just, equitable, and healthy society.

While it is recognized that “emotionally difficult experiences, including trauma, interpersonal conflicts, work stress, and social rejection, contribute to chronic pain” [29, 30] (p. 565), the systemic and structural nature of these are not well understood [31], and research is only beginning to be conducted with socially marginalized groups. For example, the unique mechanisms whereby systemic racism becomes manifest in painful experiences and how it influences the treatment of chronic pain is not well studied. In the context of low back pain, Ziadni et al. [29] found pain-related appraisals of injustices mediating these relationships, with prior experiences of racial discrimination positively associated with severity of disability and depressive symptoms. Perceived injustices have also predicted more chronic self-reported experiences of depression and disability outcomes in people with chronic low back pain [32]. Experiences of pain were linked to practices of discrimination and injustice and as these increased, individuals’ perceptions deepened regarding the irreparability of the pain they were encountering and their sense of unfairness related to their experiences of care [29].

Practices of injustice and unfairness can have negative impacts on overall health, with these adverse impacts amplified for people living with chronic pain who may increasingly perceive the care inequities they encounter and the pain they face as reflecting excessive social scrutiny, social judgement, and a form of punishment [33]. Moreover, while previous research has acknowledged systemic inequities in the distribution of pain and pain management, programmatic responses to these inequities in the form of improved access to interventions have been minimal and are understudied [22, 32]. While health inequities, including those associated with chronic pain, are well documented and calls for equity are commonplace, there is a lack of engagement and research in exploring how to address inequities within healthcare responses.

The purpose of this study was to provide first person accounts of the relationship between experiences of pain and discrimination and stigma, thereby informing equity-oriented responses to chronic pain tailored to people experiencing both pain and social marginalization. To study the relationship between chronic pain and diverse experiences of social marginalization as well as implications for interventions, we conducted a community-based qualitative study using focus groups with people living with chronic pain and identifying with one of three groups known to experience high levels of inequity, discrimination, and stigma in Canada, specifically Indigenous^1^[34, 35], LGBTQ2S^2^ [36] and refugees or newcomers [34].

We employ a health equity framework to investigate the links among the complexities of inequities, social marginalization, and structural violence to inform equity-oriented responses to chronic pain. Namely, the EQUIP Health Care program of research [37–40] informs the theoretical framework for this inquiry as well as the analytical processes. EQUIP offers an evidence-based theoretical framework that describes equity-oriented health care (EOHC) as enacting three key dimensions: trauma- and violence-informed care, culturally-safe care, and harm reduction, each of which must be tailored to the particular health care context and populations served [37]. EOHC aims to reduce the effects of structural inequities by analyzing inequities and their impact, and augmenting and providing alternatives to dominant approaches to care [38]. This analysis aims to provide direction for tailoring EOHC for those who experienced chronic pain and health inequities.

## Methods

The research was guided by the principles of community-based participatory research (CBPR), an approach that engages communities equitably in the research process for the purpose of producing knowledge and taking action for positive change [41, 42]. The research was initiated by Pain BC, a province-wide, nongovernmental organization that brings together people in pain, their families, health care providers, and others to improve the lives of people living with chronic pain in British Columbia (BC) Canada and beyond. Pain BC identified the need to develop contextually tailored responses to chronic pain for populations in BC also experiencing social marginalization.

Throughout the project, Pain BC played a lead role in the research processes and, as the principal knowledge user, is using the evidence to inform their current and future programming. The research team includes community-based researchers with unique, often longstanding, relationships with the communities experiencing social marginalization. The researchers collaborated with not-for-profit organizations in these communities to recruit participants: an Indigenous community health centre in one community, a resettlement and newcomer agency in another, and a network of agencies and contacts dedicated to LGBTQ2S communities.

Focus groups were utilized as a data collection approach that holds the potential for in-depth exploration of issues through shared discussion and understandings. With the CBPR approach, the gathering of individuals with shared experiences also offers the potential of creating community connections, building capacity about an issue and a foundation for further participation and action.

 The overall sampling approach was convenience sampling via third-party recruitment with strategies modified to best fit the community interests and processes, as well as outreach from the collaborating community organisations who linked interested participants with the researchers. Recruitment handouts were distributed by the collaborating agencies to people accessing their sites and services. Participant criteria included being an adult (over 18 years), identifying as experiencing chronic pain for three months or longer and identifying as a member of the population group being gathered (Indigenous, LGBTQ2S, or refugees and newcomers). An honorarium of $50 (CAD) was provided to each participant. Collaborating agencies continued recruiting until the handbill had reached those who might be interested and a number of people sufficient for a group (between six and fifteen, given our aim of 6–8 people per group and potential for two groups in each setting), had expressed interest in participating.

Four focus groups were held with a total of 36 participants, with the initial group conducted in December 2018 and the final group in March 2019. The Indigenous group and the LGBTQ2S group each had 11 participants, one newcomer and refugee group had six participants, while a second group was organized with 8 refugees from Syria, facilitated in Arabic and translated to English. The research team decided to not include a demographic survey as part of data collection as we considered using predetermined categories antithetical to CBPR processes and our intersectional perspective. Rather, each group began with each individual introducing themselves as they wished, which often resulted in participants presenting their complex selves and identities more fully than a demographic survey might have elicited.

Within the focus groups, we sought to create a culturally safe social opportunity with the sharing of a meal that included moose stew with the Indigenous group, Persian catering with the newcomer and refugee group and the LGBTQ2S group had lunch from a queer-based caterer in the community. The researchers worked to reduce barriers to participation and to respond to diverse access needs for people living with chronic pain including low lighting, supportive seating, specific dietary requirements and the presence of translators. The focus groups typically exceeded an hour with the meal and socializing being close to another hour.

The focus groups were facilitated by the researchers or co-facilitated with a community member including an Indigenous Elder with the Indigenous group and a settlement worker with the newcomer and refugee group. At the start of the group, consent forms were explained, notably we clarified how consent and confidentiality functions within group discussions. Following opportunities for any questions to be discussed, the consent forms were signed and collected. The same semi-structured interview guide was utilized in all groups to explore three general areas: (a) participants’ experiences of barriers (both external/systemic and personal) in managing their chronic pain, (b) what participants found helped them deal with their pain, and (c) participants’ insights on what could be improved to provide better health care. The group discussions were audio recorded and then transcribed. Ethical approval for the project was granted from the University of British Columbia Behavioural Research Ethics Board (H18-01481).

Data analysis was a thematic analysis guided by coding techniques from grounded theory [43] with transcripts analysed by the research team, including Pain BC research collaborators, using an iterative process that made use of both deductive and inductive processes. To be inclusive of collaborators the coding was conducted in our word processing programs using coloured highlighting and comments rather than using qualitative software. An initial deductive analysis was conducted using a priori categories based on the EQUIP framework: These themes were trauma and violence informed care, culturally safe care, harm reduction, and contextually tailored care [39]. Second, themes were identified inductively from within and across all focus groups. Following this process, the team brought together both inductively and deductively derived themes to develop an analytic framework as described by Corbin and Strauss [44]. A subsequent round of coding was conducted using the analytic framework. The research team once again discussed the themes as a group and collaboratively developed a more detailed thematic analysis that identified overarching themes and sub-themes within and across groups.

Within a month of the final focus group, a one-day workshop was held with an objective of sharing the initial thematic analysis with stakeholders who included two or three participants from each focus group, Pain BC, the research team, and a small number of key chronic pain healthcare providers and policy makers. This feedback process provided an additional analytical step as research participants and other stakeholders provided reactions to the findings, including their analyses of what was presented. From this step, the research team met again to refine and finalize the analytical framework and reanalyze the full transcripts.

## Results

The purpose of this study was to examine in greater depth the experiences of pain and discrimination and stigma across diverse marginalized communities in order to recommend equity-oriented healthcare approaches. The data suggests that participants’ experiences of pain were entangled with and shaped by five key interrelated areas: their social locations, identities and related barriers they faced; their experiences of violence, trauma and related mental health issues; their experiences of discrimination, stigma and dismissal; their experiences of inadequate and ineffective health care; and the impacts of these intersecting experiences, specifically on employment, income, and their social lives.

### Pain was shaped by and entwined with participants’ social locations and identities and related barriers

These diverse groups of people were brought together because of a single aspect of their social location: identifying as LGBTQ2S, identifying as Indigenous, or being a newcomer or refugee to Canada. While participants identified the ways in which they aligned with the group to which they had been invited (e.g., most newcomers specified from which country they had immigrated), they also often offered multiple dimensions of their social locations and related identities extended beyond the group they had been invited to represent. Indeed, some participants also identified with one or both of the other groups: a transwoman participated in the Indigenous focus group; several Indigenous persons participated in the LGBTQ2S focus group and so on. Notably, the Indigenous Talking Circle was guided by a protocol for introductions, and although it is common practice within such protocol to name one’s clan, community, territory, or nation, most of the Indigenous participants primarily stated their relationship to pain in their introduction. This was unusual, perhaps in part explained by the fact that the focus group facilitators (a local Indigenous Elder, and a researcher) were familiar to most and had introduced themselves formally on other occasions. Also notably, no participants in the refugee and newcomer groups shared aspects of their gender identity or sexual orientation perhaps in part because in many of the cultures of which the newcomer participants were representative, terms such as ‘gay’ or ‘trans’ may not be part of the cultural vocabulary, even when people align with those identities [45].

Across these diverse groups, participants expressed the complexity of identity and social location, and the complex relationships among them.

I’m trans, I’m non-binary, I’m Jewish, I’m a singer, I’m going through transition right now and that’s something that’s been on my mind a lot is that my voice is changing and I’ve been grieving my singing voice a little bit so just thinking about that before I got here today (LGBTQ2S-1-1).

For some, pain, and disability rights were part of their described identity. “I identify as queer and non-binary and I also identify as a crip, sick, and disabled” (LGBTQ2S 1–6). Indeed, as noted, most of the Indigenous participants opened with a declaration of their long history of pain. “My name is M and I’ve been living with chronic back pain since 1990” (Indigenous 9).

Perceived identity and social location were described by many as acting as threats to safety and as barriers to pain treatment and support. LGBTQ2S 1–6 went on to say “I’ve had a lot of interactions with the healthcare system and I’ve definitely come across times where there weren’t resources that really reflected who I was”. Another said:

As an Indigenous person I always feel like the health, in totality, the healthcare system is a barrier to me. There’s always a risk of – I always feel, I have to measure if there’s a threat against my body or even my family just because somebody could make a racist assumption about me. (LGBTQ2S 1–10)

Specific identities or social locations were associated with unique barriers to treatment and support, including specific threats to safety such as accessing gender-affirming care.

I am gender fluid, looking at taking testosterone and I’m curious about, broaching that with my doctor who’s already very binary focused and in tandem with all of the mysterious things that cause pain and mental health issues… and then the layer of just very much like not knowing if I’m safe, being seen or willing to be asked to be seen or whatever is a barrier as well (LGBTQ2S 1–7).

Participant’s social locations and identities were directly associated with specific structural barriers. For example, Indigenous participants’ access to services varied according to whether or not they had status^3^ under the Indian Act.

When I say I’m Native I have to say non-status. I have to actually specify that, that I’m non-status because… my biological father is not on my birth certificate. I’m actually persecuted by both sides, by the Native side and the Canadian, white Canadians. And I kinda get thrown around by both of them because I’m not either one. (LGBTQ2S 1–11)

Similarly, newcomers’ experiences varied with their status governed by legislation related to immigrants and refugees and the associated benefits to which they were variously entitled, with ‘undocumented’ newcomers having no access to healthcare. More generally many newcomers cited the high cost of health care as another structural impediment to accessing care. Newcomers without strong English skills described difficulty in even describing their health issues and pain, particularly given that many health care settings do not provide access to interpreters, with consequent under treatment, delayed treatment, mistreatment and emotional impact.

Emotionally it’s very hard when you can’t, when the services can’t understand you and doctors don’t understand you… (Newcomer 2 − 1).

The social identity status of being a refugee newcomer with its attendant consequences of social isolation was also identified as a cause for the advent of chronic pain.

She says before she [came to Canada], when she was in [her country of birth] she never feel anything, she was relaxed enjoying social life, meeting each other occasionally or meeting family, friends, whatever, go to church, go to mosque, whatever, she never feel that. But since she came here all the pain start to come out. (Translator for Newcomer FG2)

### Pain was shaped by violence, trauma and related mental health issues

Aside from social marginalization due to their identity locations, in all groups many associated their chronic pain with personal experiences of trauma and related mental health issues.

I’m a refugee … from [place]. I went through so many difficult things. I was raped and kicked, I was being kicked with boots, soldiers were kicking me with their boots, I don’t know how my life came to be so broken down. After that I had no peace. I came here, I was happy that I’m here…I thought again I’m going to experience joy, happiness but it’s not easy. I’m going to hospital, just to the hospital and I thought my kids were going to move and come and stay with me in this country. My kids never moved and follow me. My head is not working, I have high blood pressure, most of the time the pressure will go up to two fifty, three hundred. I’m taking drugs but the pressure does not come down. And the pain, I’m telling you… All night I’m walking (pacing) in the house while others are sleeping and I’m awake. Then I sleep in the daytime and we are supposed to live as a family and do chores and other things that means we are living as a family, but I can’t because I have not slept all night. (Newcomer 1–6)

A participant from the LGBTQ2S community also shared the following:

I live with chronic pain and had pain probably my whole life and diagnosed with learning disabilities, as well as chronic mental illness and trauma, so all those kind of go together. (LGBTQ2S 1–4)

Yet another participant identified trauma and mental health as closely related to their experiences of pain:

..in addition to like um physical kind of pain stuff, like mental health struggles and complex PTSD and trauma is something I’ve lived with most of my life as well and I think it’s been like a big source or like really important in my um, my resulting chronic pain. (LGBTQ2S 1-NS)

### Pain, discrimination, stigma and dismissal

Along with systemic barriers to accessing health care as described in the first theme above, discrimination, stigma and experiences of having their concerns dismissed by health care workers was identified as a significant and common experience across all participant groups. The participants’ experiences of pain were described in ways that were inseparable from their experiences of discrimination, stigma, and dismissal. As suggested earlier, throughout the focus group, and during the one-day workshop, many in the LGBTQ2S group described how cisnormativity and heteronormativity that pervades multiple systems influenced their experiences, and thus the meaning of pain.

…I wanted to share…about being a student and being at post-secondary institutions and experiences like working with the disability resource centres on campuses that I’ve been at and with various professors and others that are above [laughs] me as a student. And also in combination with my fibromyalgia, which is chronic and constant kind of pain but also having PMS and menstrual issues combined with the gender identity stuff makes just a whole other mixed bag of things to limit you in sort of access to education and to acceptance, yeah. (LGBTQ2S -NS)

All of the Indigenous participants described experiencing race-based discrimination, with most describing being constructed as “drug seeking” by health care workers, regardless of whether they wanted medication or not.

Well I used to use drugs…I don’t do that anymore. I’m on methadone now and I’m in recovery, so. But…because I’m on so much methadone… it’s hard for me to manage my pain…when I do go to the hospital and stuff, they look up my past history and my drug life, and …they always bring that up. It’s always like, they won’t give me the medications that I should be getting because they think I’m drug seeking. (Indigenous 1).

Participants described how such assumptions were interrelated with their experiences of not being believed which in turn deterred them from seeking and getting care:

For me, I’m having a hard time to ask for help to any doctors because of how they treat me, like [they] say I just want to get pain medication just to get high and all that, and I’m really in pain and I’m telling them and they don’t believe me and that, and I’m having a really hard time right now. It is hard for me to ask because of all the things I went through with the doctors and that. They treat me like…I’m just after the drugs to get high and that. They don’t believe me, that I’m in pain. I’m trying really hard to ask for help and it’s really hard for me to ask for help because of how I was treated before. (Indigenous 3)

These experiences of discrimination, stigma and dismissal were entwined with participants’ experiences of inadequate and ineffective health care, together shaping their understanding, experiences, and meanings of pain.

### Inadequate and ineffective health care

Overwhelmingly, most participants described being unable to find effective pain management.

Nothing has been working for pain management. I’ve tried massaging, I’ve tried IMS, I’ve tried acupuncture, I tried medicinal marijuana, I’ve tried like stronger medication, I’ve tried street stuff and nothing is making it any better. (LGBTQ2S 1–9)

I was finally diagnosed with osteoarthritis in two places in my foot and I’m presently waiting now to see the orthopedic surgeon, which … is going to be another year or year and a half before I’m getting some help there. At least now I’m getting treatment, like I have physio every Monday, I have Voltaren cream that I can put on my feet; whereas for six years, no physio, no cream, no pain medication. It just kept escalating and escalating. And I think part of the reason why it is as bad as it is now, is because there was nothing for six years. No physio, no suggested anything for it; just waiting for the next doctor. (Indigenous 7)

Most were only able to access pharmaceuticals and many in each group described this as very limited and problematic.

Finally, they put me on 2 mg of Dilaudid twice a day, once in the morning and once in the afternoon, and they told me I could go on 1 mg four times a day, and it was like wow, yeah, okay [sarcasm]. So, I take several different painkillers, but I don’t want to be on medication; I want solutions. I want to be fixed. (Indigenous 6)

A participant from another group attested to a similar experience:

…like pharmaceuticals don’t really work for me and like in my experience like very few practitioners are willing to talk about any other options and are willing to work with like other types of practitioners (LGBTQTS).

This was reiterated by a participant from the refugee newcomer group:

I go to complain to the doctor and he give me sleeping pills and I say, no, I don’t want to do that, it control me. And he always give me, give me the prescription, prescription all the time but he don’t give me the specialist doctor. (Newcomer 1–3)

As described earlier, participants recounted being disbelieved regarding the existence of pain, their level of pain and the cause of their pain, and dismissed regarding their concerns, needs and preferences, leading to ineffective health care. In each group several participants described how the impact of being disbelieved and dismissed had led to misdiagnoses and mistreatment.

Pain. I honestly don’t know what it’s like to live without pain. I had back surgery a long time ago…They wanted to put those rods in my back and I told them natives tend to reject those rods, so I don’t want to go through that. They wouldn’t listen to me. Well, at the fourth month after having them in me… I started feeling my body was rejecting the steel. I went in and I told them and they did not listen to me. They said that it was in my head, that …I was trying to get drugs. …at the year mark of them being in my body, I was rushed to emergency in [another community]… they had to open me up as soon as I got in there and there was steel and pus and everything, like large amounts… For somebody to have two big jugs of liquid pus come out of them, there has got to be something that can be done before this, and where they had those bolts, it broke the bones, it ate the bones. So, I’ve got nine bones in my body that are gone because of this one doctor ... He crippled me. (Indigenous 4)

A newcomer described a six-year quest for treatment of pain. She described being dismissed repeatedly by physicians in primary care, and not believed by other healthcare providers.

So, I told myself I need to go to emergency not [a] walk in clinic because it will take me a long time. So, when I went there, I told them that I have strong headache and I show them the place exactly the place. And I told them that I need, I need help because I can’t help all these pain. The doctor he didn’t believe me at first, he told me you just have a migraine…I told him, no, it’s not that, I feel something in my brain it push my brain away. But he didn’t believe that, he like I felt he’s just feels that I am like acting like I am….

R: You are faking it?

P: Yeah and my husband told him can you please do a CT scan, something better to, to know what she has. Like when they do the CT scan they found the tumor that took all the right side. (Newcomer 2–3)

Many experienced difficulty knowing what to do and navigating the system, reflecting how systems are not organized in ways that are tailored to needs. One newcomer described:

My doctor, after taking x-rays and, yeah, trying to figure out what it was- the first thing the doctor says is “go to a physiotherapist” and then you do that. But you have to choose the physiotherapist, you have to find out which one would be better for you and where to go – you have to do all the research. It’s not that they say, okay, this physiotherapist works with me, this doctor, and I know this one is better for this arm and this one is good for the knee, no, they don’t give you zero, nothing. And you have to do the research, you have to figure it out… (Newcomer 1–3).

The inadequacies of health care shaped the meanings of pain for participants and worked to deter access to care, and to worsen the impact of pain.

…every time I go to a new practitioner I have to like tell them my whole like life story all over again. And it’s like really exhausting…it’s like I have to be really vulnerable and if they’re not trauma-informed it’s just an awful experience, to be that vulnerable they’re kind of just like seeing things through a certain lens and, yeah, I don’t feel believed or like I’m going to get, like the options they present aren’t going to be like what I’m looking for. (LGBTQTS)

### The impact of chronic pain: Complex and multifaceted

Participants described the effects of chronic pain in ways that demonstrated how the experience and impacts of pain emerged from the complex intersections among the emotional, mental, physical, social and economic dimensions of their lives as they endured the consequences of multiple forms of stigma and discrimination and structural barriers across systems. However, participants also exhibited resilience and worked to navigate significant systemic barriers to adequate care. For example, one newcomer continued her quest to improve her English in order to deal with a cascade of events stemming from inadequate interpretation.

…it’s a very hard way because the government they took away my kids – fifteen years I have no kids, once a year, one hour, they only let me visit my kids, it’s hard. And it’s because of how the interpreter translated, all I said when I was at the psychiatrist office is that my life is so hard that it looks like it’s better if I die. But that does not mean I wanted to kill myself, I was just trying to say that my life was so hard that’s all. Life not easy for me, die better than to live. But the interpreter they say I want to kill myself … and after they took me, put me in an asylum for four years. They destroyed my life, I’m so upset and I say I’m not crazy, I’m normal, I’m not crazy and I don’t want to go there, but they put me in and gave me a lot of pills, a whole bunch, and they put me in and that’s why I’m very upset. And I said, no, I stop to go to counsellor, I stop going to anything and I say, no way, until when I can speak by myself (in good English) okay? (Newcomer 1–8)

Specifically, across groups, participants emphasized impacts of the intersections of pain and barriers to support on their mental health, employment, income, and their social relationships:

I come from my country, I run away from the war, I came here but now see…. I just want to be human, I want to be like normal people. I don’t want to be like people who stay inside the home feeling scary, scary all the time. (Newcomer 1–5)

…my experience of living with chronic migraine is the most limiting of the things that I live with and it’s caused really intense social isolation which isn’t the greatest for my mental health. (LGBTQ2S 1–8)

The effects on employment included enduring additional pain during work, the challenges of fulfilling work expectations, and for many, being unable to get or continue working.

I have been in pain for well over 20 years so I’ve kind of learned to just grit my teeth and cope kind of thing. I was working. My job was really, really important to me [sigh]. So, I went from working eight hours a day down to six hours, down to five hours, to four hours, and I had to leave my job about three weeks ago because I just, I couldn’t even get in a two-hour shift anymore. (Indigenous 6)

I do nails as a job but now I can’t hold the hand to do nails, so I have to stay home. I can’t work (Newcomer 1–3).

…in relation to chronic pain I’ve been [a specific service provider] and actually just had to, at the end of last year, had to give up my license because I can’t afford to carry on that profession anymore. (LGBTQ2S 1–8)

The economic impact was profound across the groups, both as a consequence of the impact on employment, and as a consequence of the costs of support and treatment.

I’ve had pain since childhood. I became a lot more disabled by it in my early twenties and have been going to school, in quotation marks, part-time to survive, because I can’t work and now I can’t go to school. (LGBTQ2S 1–7)

Many simply could not afford treatment, even when they found modalities that were effective. A newcomer who had debilitating pain post surgery and chemotherapy for a brain tumour had to stop accessing treatment; the situation was exacerbated because they did not have proper documentation to stay in the country and therefore were not eligible for health care benefits. “…it was paid from, from me and I just didn’t have any more money and I had to stop going”. (Newcomer 1–2).

I had a referral to the podiatrist…so I go to make an appointment and everything and then the podiatrist says you have to pay $50 up front …I’m like what? I’m on disability, plus I have status. So, the lady at the reception…said well you can get a form filled out from the Ministry to get it paid for. So I go through all the trouble to get that done, call to book an appointment telling them that I have the form filled out so the Ministry can pay for it, well you have to still come, bring $35 cash to pay for the filing fee…So I never did see the podiatrist because it just wasn’t affordable. (Indigenous 6)

Participants’ experiences of chronic pain were described in relation to others, including family and community members, both in terms of how others influenced their experiences and how their chronic pain affected others.

I’ve spent more than half of my life being in severe pain. I’ve had fibromyalgia for 26 years and I had to relocate because of it. When we lived in (another community) I spent a lot of time in the hospital. My marriage ended because of my health problems. I had a very spoiled husband who for the first four years of our marriage came home and his dinner was made, he didn’t have to lift a finger to help, and then when I got sick he had a hard time with it. (Indigenous 6)

Many (mostly women) were caregivers. For example, one newcomer described the impact of caring for her daughter, and how pain in turn affected her caregiving and their wellbeing:

I have a little girl in a wheelchair and she doesn’t walk, is non-verbal. She’s not such a little girl and she’s getting heavy. This has been going on and on and on for so many years – she’s fourteen. So, it’s many years of lifting but now instead of being both sides of my body now I was completely restricted in one and its really damaged… And the stress from [paying for treatment] and the stress of having to spend more money on my daughter than me because you always put others first. (Newcomer, 1–1)

Another woman was at a loss regarding how to manage in the face of her inability to work, her debilitating pain and the need to care for her children on her own.

I’m stuck and I’m stuck and that’s why I try. I try to go to school (for upgrading skills) but I cannot go to school because I have three children. I’m a single mum with three children …I ran away from [an abusive husband] that’s why I have to be careful for my children (Newcomer 1–5).

## Discussion

The purpose of this study was to examine in greater depth the experiences of pain and discrimination and stigma across diverse marginalized communities to inform and tailor equity-oriented responses to chronic pain so that people receive respectful, just, and effective care. Through these focus groups we heard how the experience of pain for people who face systemic inequities and injustices emerges from the complex intersections of the broader social environment. Chronic pain is as much a social issue as it is a health issue. For participants in the study groups, social locations engendered specific forms of social inequities, dismissal, discrimination and stigma which shaped their experiences and meanings of pain. People described experiencing harms on the basis of their social identities by health care professionals and others in the community. Overall, participants’ experiences of pain were deeply informed by a composite of experiences that included discrimination, stigma, dismissal and social and economic disadvantage as well as experiences of trauma. They reported “not being listened to nor believed” by care professionals and others and “violated” by the stigmatization, stereotyping, racism, sexism, heterosexism, cisnormativity, and transphobia they faced in the health care system [18]. These experiences of injustice were compounded by poverty and inadequate access to supports and resources. Treatment of pain was particularly defined by multiple and intersecting forms of systemic violence and discrimination.

Thus, participants’ pain was shaped by others, particularly health care providers and broad social systems related to health care, housing, immigration and income, and people’s positions within those systems. Fortunately, there is increasing attention being paid to the impact of privilege and disadvantage on experiences of pain and access to care, for example, in terms of race [23], sexual orientation [46] and gender [40]. In particular, we identify the deleterious impact of care provider actions that the participants experienced as unjust and unfair. When compared with non-minority counterparts, pain-related injustice appraisals are elevated among those who identify with marginalized minority racial/ethnic groups and among individuals with fewer socioeconomic resources [32, 47].

Here we review the findings from the perspective of the Equip Health Care framework in our pursuit to inform equity-oriented care responses to chronic pain for people facing health inequities. We utilize the framework by discussing the findings according to the EQUIP framework’s three key dimensions: trauma- and violence-informed care, culturally-safe care, and harm reduction, each of which must be contextually tailored [37].

Analysis of the impact of these key dimensions [48] suggests their potential for explicit adaption to enhance equity oriented responses to chronic pain. In this investigation, these key dimensions were studied in primary care clinics serving highly marginalized populations. Chronic pain was measured using von Korff’s 7-item chronic pain scale [49], assessing pain disability in the past 6 months. The project introduced a new measure of equity‐oriented health care: the Equity‐Oriented Health Care Scale (E‐HoCS). Using longitudinal data from a cohort of 395 patients facing systematic inequities, the study demonstrated that more equity-oriented health care predicted better health outcomes over time, as such care was associated with higher levels of patient comfort with their providers as well as confidence in their ability to manage and prevent health problems including chronic pain [48]. Equity-oriented care predicted less disabling chronic pain as well as better quality of life and fewer depressive and trauma symptoms [48]. Further, an intervention integrating these key dimensions in primary care was able to increase staff confidence and comfort in providing such care [38]. Hence, this approach may be useful to promote equity-oriented care in relation to pain, and support people facing the greatest stigma, discrimination, and structural barriers toward improved outcomes.

***Contextually tailored care*** customizes the three key dimensions of equity-oriented care to ensure responses are suitable to the populations served and local contexts [38, 50–52]. Although described separately, the three dimensions are interconnected in practice and need to be tailored to each particular context: in this case, people experiencing chronic pain and multiple intersecting forms of social marginalization. The objective is to provide responses beyond the individually focused concept of patient-centered care to orient and adapt services, policies and practices to be optimally responsive and respectful to both those being served and the local, social, and community contexts in which care is provided.

***Trauma-and-violence informed care (TVIC)*** expands trauma-informed care to also be explicitly responsive to the structural and interpersonal forms of violence that most people affected by systemic inequities experience [53–55] and that can be seen in our data. Providers seek to create safe environments and foster trusting relationships that build on their awareness of the pervasiveness of trauma and violence and its impacts on health and well-being. Being responsive to current and past trauma and violence includes attention to structural violence, as well as individual and interpersonal experiences of trauma. Providing TVIC to people living with chronic pain includes understanding the history and context of individuals’ lived experiences, recognizing trauma and violence as a risk factor for pain and pain outcomes, and adopting this strategy as a universal provision. The building of trust and respect can begin with recognition of one’s power as a provider and the impact that marginalised social identities can have on the meaning-making of pain for people living with chronic pain. Pain management would include a respect for the ways that people cope with trauma and violence, its links to mental health and substance use, and fostering trust by incorporating safety within care plans.

***Culturally-safe care*** is guided by a deep understanding of the current and historical impacts of inequitable power relations, racism, colonization, marginalization and criminalization of people based on race or ethnicity. Rather than attending to differences based on race and culture as is characteristic of most cultural sensitivity and competence approaches, cultural safety places responsibility on care providers to create safety, including challenging discriminatory values and assumptions. Because equity requires care according to need, then, for example, racialized newcomers with limited official language skills would be accorded the support required to navigate pain care systems. Because cultural safety requires addressing racism, the pernicious stereotypes directed toward Indigenous people that deter health care access and quality [56–59] would be explicitly addressed in relation to chronic pain. Cultural safety is also a framework adapted to LGBTQ2S health [60] as well as to people who use drugs [61–63], among others.

***Harm reduction*** includes the philosophy, policies and practices that seek to prevent the harms related to substance use rather than reducing substance use per se [64]. Substance use is viewed as part of society and life, and a health issue. Harm reduction approaches challenge policies and practices that cause unnecessary harm including the harms of inequities, criminalization of drugs and drug use, and refusing care to people who use substances [65]. It is an approach that provides pragmatic supports while avoiding judgement and expressing compassion for the dignity of each person. Stigmatizing certain substances and people who consume them causes harm and barriers to care. Substance use and chronic pain are linked and understanding, respecting, and responding to these links are integral to both providing care that is respectful and to building trust [66]. Respectful engagement avoids stigmatizing language and mistrust of people with pain as “drug seeking” while openly supporting individual’s own harm reduction practices by providing harm reduction supplies and ensuring signage conveys respect rather than zero tolerance (intolerance) [57].

These key dimensions of equity-oriented care can be seen as more than ideals. The Equip framework seeks to operationalize these dimensions by identifying strategies for enhancing capacity for equity-oriented services [37]. These strategies are recognized as intersecting and flexible enough to be tailored to both local contexts as well as for various health inequities such as chronic pain. Overall, these strategies are relevant to support equity-oriented responses at the organizational, clinical programming, and patient-provider interaction levels [37]. Organizationally, the strategies have the potential to improve the ‘fit’ between people’s needs and services, increase trust and engagement of clients, and shift from crisis-oriented care to continuity of care [37].

While many of the strategies for equity-oriented care are inexpensive to implement, the uptake of EOHC requires more than strategies to improve access to care. There is a requirement to question the meaning of care and the meanings of care held by people experiencing inequities. How responses to chronic pain are arranged and delivered matters to people experiencing inequities. Providers and organizations require a foundational understanding of and commitment to equity-oriented care. Indeed, disruption should be expected and embraced if organizations are to shift meanings of care to more fully reflect equity-oriented dimensions [38]. Such productive disruption may be essential to move beyond the ongoing measuring of inequities and well-intentioned calls for health equity [38].

The scope of inequities in health overall and chronic pain specifically are well established with ample recommendations to improve access and outcomes for people who experience inequities and injustices. Further, while the relationship between perceived injustice and chronic pain outcomes has been acknowledged, there remains a paucity of equity-informed interventions that explicitly address inequity at point of care.

Building on the findings from this and other studies, equity-oriented responses to chronic pain would establish pain not only as a health care issue but as a social justice issue. The meanings of pain and power would be inseparable and addressing pain would be addressing privilege and power within system responses to pain. Addressing pain would be inclusive of identifying and challenging the inequities and injustices that are producing and sustaining the stigma, discrimination, powerlessness, and often violence experienced by those also experiencing pain [27]. For example, based on their findings regarding racial differences in chronic pain experiences and outcomes, Trost et al. [32] call for systems-level interventions that go beyond individual level interventions to address possible antecedents to elevated injustice appraisals both within medical contexts and broader social structures.

## Limitations

There are a number of limitations to this study. The small number of participants cannot be expected to capture the full diversity among the groups represented nor the complete range of experiences and perspectives. The recruitment locations influenced who was able to participate. For example, the Indigenous focus group was recruited from and held in a clinic which also houses a methadone clinic and thus a number of participants reflected these lived experiences. We expect that the study appealed to those who may have experienced the most challenges and were most interested in discussing these issues. At the same time, we are aware that the impacts of chronic pain limited some interested individuals from fully participating or attending at all. Significant accommodations were necessary to reduce barriers to participate but we were aware that some individuals regretted not being able to participate. Finally, as a community-based research project we recognize that community participation varied within the research process [67] with greater inclusion in some aspects and less participation in others.

## Conclusions

This study underscores the complexity of the experiences and meanings of pain for people living with pain and facing social disadvantages, stigma, discrimination and structural barriers to support such as poverty, systemic racism, sexism, cisnormativity and heteronormativity that are embedded in and supported by policies and social arrangements. This analysis emphasizes the inadequacies of an exclusive biomedical orientation to the understanding and treatment of pain, in particular the over-reliance on pharmaceutical management, and points to the need for efforts toward equity in the response to pain. Equity requires treating people according to needs, not treating everyone the same. The specific experiences of these participants highlight the connections among pain, mental health and substance use and shows how stigma, discrimination and dismissal of the meanings and experiences of people living with pain must be tackled through change, not just by care providers, but by innovations in systemic structures. The EQUIP Framework is an approach to integrating trauma- and violence-informed care; culturally-safe care; and harm reduction in health care that may hold promise for being tailored to people experiencing pain and social marginalization.

## Data Availability

The datasets used/or analyzed during the current study are available from the corresponding author on reasonable request.

## Footnotes

1 In Canada, Indigenous people include diverse First Nations, Inuit and Metis peoples. Royal Commission on Aboriginal Peoples. (1996). *Report of the Royal Commission on Aboriginal Peoples*. Ottawa: Canada Communications Group-Publishing).

2 Lesbian, Gay, Bisexual, Transgender, Transsexual, Queer, Questioning and Two-Spirit (LGBTQ2S).

3 Indian status is the legal status of a person who is registered as an Indian under the Indian Act 46.Act I. Indian Act, RSC 1985, c I-5. 1876.. The Indian Act is a race-based act of legislation that categorized Indigenous persons and governed all aspects of their lives, and remains in force today, with a few revisions since 1885.
